# Autoantibodies in Systemic Sclerosis: Unanswered Questions

**DOI:** 10.3389/fimmu.2015.00167

**Published:** 2015-04-15

**Authors:** Cristiane Kayser, Marvin J. Fritzler

**Affiliations:** ^1^Rheumatology Division, Escola Paulista de Medicina, Universidade Federal de São Paulo, São Paulo, Brazil; ^2^Faculty of Medicine, Cumming School of Medicine, University of Calgary, Calgary, AB, Canada

**Keywords:** systemic sclerosis, scleroderma, autoantibodies, anti-nuclear antibodies, autoimmune diseases

## Abstract

Systemic sclerosis (SSc) is an autoimmune disease characterized by vascular abnormalities, and cutaneous and visceral fibrosis. Serum autoantibodies directed to multiple intracellular antigens are present in more than 95% of patients and are considered a hallmark of SSc. They are helpful biomarkers for the early diagnosis of SSc and are associated with distinctive clinical manifestations. With the advent of more sensitive, multiplexed immunoassays, new and old questions about the relevance of autoantibodies in SSc are emerging. In this review, we discuss the clinical relevance of autoantibodies in SSc emphasizing the more recently published data. Moreover, we will summarize recent advances regarding the stability of SSc autoantibodies over the course of disease, whether they are mutually exclusive and their potential roles in the disease pathogenesis.

## Introduction

Systemic sclerosis (SSc) is a chronic autoimmune rheumatic disease (ARD) of unknown etiology characterized by microvascular abnormalities, cutaneous and visceral fibrosis all accompanied by signature immune abnormalities. Clinically, SSc is a heterogeneous disease with a broad range of manifestations, including Raynaud’s phenomenon, and cutaneous, gastrointestinal, renal, cardiovascular, and pulmonary involvement. SSc patients are classified as diffuse cutaneous SSc (dcSSc), characterized by widespread and rapidly progressive skin thickening, and earlier and more severe internal organ involvement, or limited cutaneous SSc (lcSSc) in which skin thickening is restricted to the distal extremities and face, and accompanied by less severe and later onset internal organ involvement (1).

The presence of serum autoantibodies directed to multiple intracellular antigens is a serological hallmark of SSc. In SSc, these autoantibodies are present in more than 95% of patients and are helpful biomarkers for establishing an early and accurate diagnosis (2). It is intriguing that they are also associated with distinctive clinical subsets, specific patterns of organ involvement, and different prognostic features (3). Some of them are considered highly specific for SSc, including anti-topoisomerase 1 (ATA), anti-centromere (CENP), and anti-RNA polymerase III, and these were recently added to the 2013 American College of Rheumatology/European League against Rheumatism (ACR/EULAR) SSc classification criteria (4). In addition, several other autoantibodies can be detected, which include anti-U3-RNP/fibrillarin, anti-Th/To, and anti-RNA-polymerase I and II that often produce a nucleolar staining by indirect immunofluorescence (IIF), and anti-U11/U12 RNP (2). The sensitivity and specificity of these autoantibodies varies in SSc cohorts depending on the ethnicity and the geographic region of origin, immunogenetic markers, as well as the autoantigen and immunoassay used (2, 5). Autoantibodies that are reported in other ARD can also be present in SSc, including anti-PM/Scl, anti-Ku, anti-U1-RNP, anti-SS-A/Ro60, and anti-NOR 90 (Table 1). More recently, some autoantibodies with possible pathogenic properties, including anti-platelet-derived growth factor (PDGF) receptor antibodies, anti-endothelial cells antibodies (AECA), anti-fibroblast, anti-angiotensin type 1 receptor (AT_1_R), and endothelin-1 type A receptor (ET_A_R) (3, 6, 7), have been described.

**Table 1:** **Frequency and clinical correlations of systemic sclerosis (SSc) autoantibodies**.

	% Frequency in SSc	Disease subtype	Clinical associations	Prognosis
Anti-centromere	20–38	lcSSc	Pulmonary arterial hypertension	Better prognosis
Anti-topoisomerase I	15–42	dcSSc	Pulmonary fibrosis	Worse prognosis
			Heart involvement	
Anti-RNA polymerase III	5–31	dcSSc	Renal crisis	Increased mortality
			Tendon friction rubs, synovitis, myositis, joint contractures	
Anti-U3RNP (fibrillarin)	4–10	dcSSc	Renal crisis and cardiac involvement	Poor prognosis especially in African-Americans
Anti-Th/To	1–13	lcSSc	Pulmonary fibrosis and renal crisis	Poor prognosis
Anti–U11/U12 RNP	3.2	–	Raynaud’s phenomenon	Increased mortality
			Gastrointestinal involvement	
			Lung fibrosis	
Anti-U1-RNP	2–14	lcSSc	Raynaud’s phenomenon, puffy fingers, arthritis, myositis, overlap syndrome (i.e., MCTD)	Better prognosis
Anti-PM-Scl	4–11	Overlap with polymyositis	Raynaud’s phenomenon, arthritis, myositis, pulmonary involvement, calcinosis, and sicca symptoms	Better prognosis
		lcSSc	
Anti-Ku	2–4	–	Myositis, arthritis, and joint contractures	–
Anti-hUBF (NOR 90)	<5	lcSSc	Mild internal organ involvement	Better prognosis
Anti-Ro52/TRIM21	15–20	Association with other autoimmune diseases	Older age onset, pulmonary fibrosis	–

*dcSSc, diffuse cutaneous SSc; lcSSc, limited cutaneous SSc; MCTD, mixed connective tissue disease; RNP, ribonucleoprotein; TRIM, tripartite motif*.

This review will focus on the clinical relevance of autoantibodies in SSc with particular attention to recently published data. Moreover, some unanswered questions will be discussed including the stability of these antibodies over disease course, if SSc autoantibodies are really mutually exclusive and their roles in the disease pathogenesis.

## Autoantibodies in SSc

*Anti-topoisomerase I antibodies* (ATA) were initially named anti-Scl-70 because they reacted with a 70 kDa protein on immunoblots. It was later recognized that Scl-70 was a misnomer because additional studies showed that this was a breakdown product of the full-length 100 kDa protein. ATA have been reported in 15–42% of SSc patients, with a specificity ranging from 90 to 100% (2, 8). ATA are strongly associated with dcSSc and a poor prognosis. Nonetheless, ATA have also been reported in patients with lcSSc and other ARD. SSc patients with ATA have a higher risk of having severe pulmonary fibrosis and cardiac involvement. ATA has been also associated with joint involvement, tendon friction rubs, and presence of digital ulcers (2, 8, 9). The association with renal crisis has been reported but not consistently found in all SSc cohorts. Moreover, the presence of ATA in patients with Raynaud’s phenomenon is predictive in that they are associated with a high risk of developing of SSc (9).

*Anti-centromere antibodies* (CENP) were first described in 1980 when tissue culture cells, such as HEp-2 cells, were replacing cryopreserved tissue sections as the substrate of choice for ANA IIF testing. A number of CENP proteins (CENP-A, CENP-B, CENP-C, and several others) have been described, but CENP-B is thought to be the primary target of the B cell anti-CENP response in SSc (2). Besides the detection by IIF on HEp-2 cells, ELISA, line immunoassay (LIA), and addressable laser bead immunoassay (ALBIA) diagnostic platforms have been adopted for the detection of anti-CENP (2, 8). There is general consensus that anti-CENP is the most common autoantibody detected in SSc cohorts with frequencies that range from 20 to 38% (2, 5, 10). Although anti-CENP are relatively specific for SSc, they have also been reported in systemic lupus erythematosus (SLE), primary biliary cirrhosis, Sjögren’s syndrome, and in patients with Raynaud’s phenomenon. As with ATA, anti-CENP are highly predictive of impending SSc in patients with Raynaud’s phenomenon (2, 9). Anti-CENP is classically associated with lcSSc and a better prognosis compared to other SSc-related antibodies. Anti-CENP is also negatively associated with cardiac and renal involvement. By contrast, anti-CENP is associated with a higher risk of pulmonary arterial hypertension (PAH) and an attending higher risk of mortality among this particular clinical subset of patients (9).

*Anti-RNA polymerase I, II, and III antibodies* (anti-RNAP) were first described in the early 1990s. Anti-RNAP I and III antibodies almost always coexist and are considered highly specific for SSc (2). Anti-RNAP II antibodies are also found in SLE and overlap syndrome, and are not as specific for SSc. The nucleolar speckled IIF pattern typically associated with anti-RNAP is not a sensitive method for the detection of these autoantibodies, and ELISA and LIA are now commonly used for their detection (2, 11). The frequency of anti-RNAP I and III varies from 5 to 31% of SSc patients. In a recent meta-analysis, the overall pooled prevalence of anti-RNAP III was 11% (11). RNAP autoantibodies are associated with dcSSc and a higher risk for renal crisis. These patients might also have a higher risk of tendon friction rubs, synovitis, myositis, joint contractures, and risk of developing a malignancy. Despite the prevalence of renal involvement, the survival rate in patients with anti-RNAP is better than in patients with ATA or anti-U3RNP (9).

*Anti-fibrillarin (or anti-U3RNP)* antibodies recognize a highly conserved 34-kDa basic protein of the U3 small nucleolar ribonucleoprotein (U3RNP) macromolecular complexes called fibrillarin. They were initially associated with a clumpy nucleolar staining IIF pattern on HEp-2 cells but their presence should be confirmed using complementary assays such as immunoprecipitation of radiolabeled proteins, specialized Western blots, or LIA (2). Anti-U3RNP antibodies are detected in 4–10% of SSc patients, and are considered relatively specific for SSc, and mutually exclusive from CENP, ATA, and anti-RNAP (2, 10). They are more frequently found in African-American patients than in Caucasian or Asian SSc patients. Anti-U3RNP are associated with dcSSc, frequent visceral involvement, and especially renal and cardiac involvement. In African-American patients, anti-fibrillarin antibodies are associated with severe pulmonary disease, pulmonary hypertension, severe small bowel involvement, and a poor prognosis (9).

*Anti Th/To antibodies* typically produce a homogenous nucleolar IIF staining pattern on HEp-2 substrates. Anti-Th/To antibodies primarily bind to two proteins of the mitochondrial RNA processing (MRP) and the ribonuclease P complexes. They are present in 1–13% of SSc patients, are considered to be relatively specific for SSc, are primarily associated with lcSSc, and a high frequency of pulmonary fibrosis and scleroderma renal crisis and, hence, a poor prognosis (9). Identification of clear-cut clinical associations has been hampered to date by the limited availability of immunoassays (8).

*Anti-U11/U12 RNP antibodies* have been described in 3.2% of SSc patients. In a study by Fertig et al (12), anti-U11/U12 RNP antibodies were associated with Raynaud’s phenomenon, gastrointestinal involvement, severe pulmonary fibrosis, and a higher risk of mortality (12).

### Other autoantibodies

Other autoantibodies, including anti-U1-RNP, anti-PM-Scl, anti-Ku, anti-Ro60/SS-A, anti-Ro52/TRIM21, and anti-NOR 90 tend to be found in SSc-overlap syndromes as well as in other ARD, and are considered less specific for SSc (Table 1) (2).

## Stability of Autoantibodies in SSc Over Disease Course

The titers of most SSc-related autoantibodies are considered to remain relatively stable over the course of the disease. Nonetheless, immunoassays that are able to reliably quantitate differences in SSc autoantibody levels have recently become available. Hence, studies evaluating the dynamics of autoantibody titers, or profiles over the protracted clinical course of SSc, or the impact of treatment have yet to be validated in multi-center studies.

Using semi-quantitative immunoassays, early studies reported that anti-CENP levels were relatively stable over long-term clinical observation (8, 13). Although controversial, others studies have indicated that increased levels of ATA were associated with the development of serious organ involvement or more prominent skin thickness (8, 14, 15). Interestingly, one study observed that ATA became undetectable in 20% of ATA positive patients during the disease course. These patients presented with less extensive skin and lung involvement and better survival rates than patients with persistently elevated ATA (15). This remarkable decrease in ATA levels was accompanied by a reduction in isotype expression and epitope reactivity (15). An analysis of 212 SSc patients who were ATA positive and had different skin thickness progression rates (STPRs) also showed a positive correlation between the immunoglobulin G (IgG) ATA levels and the STPRs and the modified Rodnan skin thickness score, suggesting that serum levels of ATA reflect the severity of skin sclerosis in patients with SSc (14).

The levels of anti-RNAP III antibodies were analyzed in two longitudinal studies but the clinical usefulness of their quantitative measurement remains unclear (16, 17). First, anti-RNAP III levels were measured by ELISA in more than 500 SSc patients although serial evaluation was performed in only six anti-RNAP III positive SSc patients (16). In four patients, the anti-RNAP III antibody level increased early in the disease course and then decreased, correlating closely with the modified Rodnan skin thickness score. Two patients developed renal crisis following rapid increases in the anti-RNAP III levels (16). In a second study of 64 SSc patients, there was no association between changes in anti-RNAP III levels and organ complications, but there was a weak but significant correlation between change in modified Rodnan skin score and anti-RNAP III levels (17).

Because of the limited data available, larger, multi-center, prospective studies are needed to evaluate the relevance of serial measurements of autoantibodies in SSc patients.

## Are SSc Autoantibodies Mutually Exclusive?

As discussed above, with the exception of Ro52/TRIM21 autoantibodies, the most common autoantibodies observed in SSc sera are considered to be mutually exclusive. However, with the more recent advent of multiplexed immunoassays, such as LIA, ALBIA, and now, the chemiluminescent immunoassay (CIA), this perspective is changing (2).

In several studies using IIF, immunoprecipitation, and immunodiffusion, only a very small proportion of SSc sera were found to have more than one SSc-related antibody (18, 19). In a study of 180 SSc patients using IIF, immunodiffusion, and immunoblot for the detection of ATA and ACA, coexisting ACA and ATA antibodies were found in 10 sera by all three methods. These patients presented heterogeneous clinical manifestations and 6 of the 10 patients had dcSSc (18). More recently, the coexistence of CENP autoantibodies and ATA was analyzed in 4,687 patients from the EUSTAR database. Twenty-nine patients (0.6%) were documented double-positive for both ATA and ACA. Sera of 14 patients were available for central reanalysis by IIF, enzyme immunoassay, and immunoblot to confirm antibody status, of which eight were confirmed to contain both autoantibodies. The prevalence of cutaneous and visceral manifestations in double-positive patients was similar in comparison to ATA single-positive patients (19).

However, in a recent analysis of 528 SSc sera from the Australian Scleroderma Interest Group (ASIG) in which 16 autoantibodies were detected by LIA, the presence of monospecific autoantibodies was surprisingly less common than the detection of multiple antibodies. For example, anti-CENP was associated with other autoantibodies in 60% of positive sera, anti-Ro-52/TRIM21 in 94%, and ATA in 39% of sera (2).

In an Italian cohort of 210 SSc patients in which a commercially available LIA for the simultaneous detection of 13 SSc-associated autoantibodies was used, with the exception of anti-Ro52/TRIM21 (specificity of 50%), all the autoantibodies were very specific (from 93.3% of anti-PMScl-75 to 100% of anti-PDGFR, AFA, and anti-RP-11) for SSc (5). Excluding anti-Ro52/TRIM21 from the evaluation because it was considered not specific for SSc, anti-PMScl-75 was associated with ACA in seven sera, with other autoantibodies in seven additional sera, and antibodies directed to both PMScl-75 and PMScl-100 were found in nine patients. Anti-Ku was associated with another autoantibody in 4 of 10 positive patients, and anti-PMScl-110 was also found associated with another autoantibody in 3 of 14 patients (5).

In another recent study in which 13 antibodies were measured by ALBIA, the simultaneous presence of at least three antibodies was found in only 4% of SSc patients, but the simultaneous presence of two antibodies was found in 17% of patients (20).

Thus, with an increasing number of diagnostic assays for the detection of autoantibodies related to SSc and other ARD, the coexistence of autoantibodies in SSc patients have been more frequently described although the clinical and pathological significance of these findings are still not clear. Moreover, more diligent attention needs to be given to the standardization of these immunoassays (5).

## Are Autoantibodies in SSc Pathogenic?

Activation of the cellular and humoral immune systems has been clearly implicated in the pathophysiology of SSc. As discussed above, although the autoantibodies present in SSc sera correlate with distinct clinical phenotypic subsets, as well as disease severity, and the risk of specific organ complications, their pathogenic relevance is still unclear. Because the vast majority of SSc autoantigens described to date are apparently intracellularly sequestered and presumably not accessible to circulating autoantibodies, there are controversies about how these autoantibodies could bind to their cognate autoantigens, and cause cell damage or related disease features (21).

Nonetheless, an intriguing pathogenic role, especially for ATA, has been suggested by some studies (21, 22). In particular, purified ATA from SSc sera were found to bind directly to the cell surface of fibroblasts, a plausible cellular target in SSc pathogenesis (22). In another study, the same group reported that the binding of ATA to fibroblasts could subsequently stimulate adhesion and activation of monocytes *in vitro* (23). Although these findings do not necessarily implicate ATA in the pathogenesis of the disease, it is an intriguing link to increased immune responses and fibrosis in SSc (6).

More recently, autoantibodies directed against non-nuclear autoantigens described in SSc were recognized as having potentially pathogenic roles in vascular damage and tissue fibrosis in SSc (2). They include antibodies directed to endothelial cells, fibrillin-1, fibroblasts, against matrix metalloproteinases (MMP), such as MMP-1 (interstitial collagenase) and MMP-3 (stromelysin), and more recently, the PDGF receptor, the angiotensin II type 1 receptor (AT_1_R), and ET_A_R (Table 2) (2, 5, 6, 24).

**Table 2:** **Pathogenic autoantibodies in systemic sclerosis (SSc)**.

	% Frequency in SSc	Pathogenic roles in SSc
Anti-fibroblast	26–58	Induce fibroblast activation *in vitro*
Anti-fibrillin-1	>50	Activate fibroblasts *in vitro*
		Stimulate release of TGF-β in the ECM
Anti-matrix metalloproteinases (MMP) -1 and -3	49–52	Inhibit MMP collagenase activity
		Reduce of ECM turnover
Anti-endothelial cells	44–84	Activation of endothelial cells apoptosis *in vitro*
Anti-PDGF receptor	33–100	Activation of the PDGF receptor
		Stimulation of reactive oxygen species and collagen production, and converting resting fibroblasts into activated myofibroblasts
Anti-angiotensin type 1 receptor and endothelin-1 type A receptor	82–83	Stimulation of the production of ROS and collagen by fibroblasts

*ECM, extracellular matrix; MMP, matrix metalloproteinase; PDGF, platelet derived growth factor; TGF, transforming growth factor*.

Anti-fibroblast antibodies have been shown to be present in up to 58% of SSc patients and induce fibroblast activation *in vitro* (25). In another study, fibroblasts stimulated with fibroblast antibodies showed an increased capacity to degrade collagen matrix and produce MMP-1 while the production of total collagen, type I collagen, and tissue inhibitor of metalloproteinase-1 (TIMP-1) was unaffected (26).

Anti-fibrillin-1 antibodies have been reported in >50% of SSc patients and activate fibroblasts and stimulate release of TGF-β (24). Anti-fibrillin-1 antibodies have shown to activate normal fibroblasts *in vitro*, resulting in increased production of collagens and other extracellular matrix (ECM) components characteristically overexpressed in SSc fibroblasts (6). Neutralization of TGF-β with anti-TGF-β antibodies significantly decreased the activation of fibroblasts by anti-fibrillin-1 antibodies. Nonetheless, their pathogenic role remains uncertain as another study, which evaluated autoantibodies against “properly” folded fibrillin-1, did not find these autoantibodies in sera from SSc patients (27).

Antibodies to MMP-1 and MMP-3 reported in the sera of SSc patients were considered to be specific for SSc and were proposed to prevent the degradation of excessive collagen and ECM components in SSc (6, 24).

Anti-endothelial cells antibodies were reported in 44–84% of SSc patients (24) and were associated with more severe vascular involvement. AECA from SSc patients have been reported to induce EC activation and apoptosis *in vitro* and to induce the caspase 3 apoptotic pathway by both ATA and CENP positive SSc sera (28). Moreover, increased gene expression of fibrillin-1 was observed in human dermal endothelial cells stimulated with SSc sera. Immunohistochemistry studies of human dermal endothelial cells also demonstrated the aberrant expression of fibrillin-1 protein in apoptotic endothelial cells treated with SSc sera containing AECAs (28).

A bioassay consisting of embryonic fibroblasts with or without PDGFR subunits detected anti-PDGF receptor antibodies in 100% of SSc patients (29). IgG autoantibodies from SSc were found to activate the human PDGF receptor and induce the Ha-Ras-ERK1/2 pathways and reactive oxygen species (ROS), which stimulated type I collagen-gene expression and also converted resting fibroblasts into activated myofibroblasts. In addition, autoantibody activity was abolished in control experiments after preincubation of immunoglobulins with recombinant PDGF receptor and preincubation of mouse embryonic fibroblasts with PDGF receptor tyrosine kinase inhibitors, suggesting that anti-PDGF receptor antibodies could be potentially pathogenic (29). However, the specificity of anti-PDGF antibodies is uncertain as they were also detected in chronic graft-versus-host disease and in healthy sera (2). More recently, agonist activity of PDGF receptor was not detected in SSc sera (2). While these studies show great promise in an understanding of the pathogenesis of SSc, the prevalence, specificity, and the role of these autoantibodies remain to be confirmed (6).

Autoantibodies directed against AT_1_R and ET_A_R were identified in most SSc sera and were associated with pulmonary fibrosis, PAH, and with higher mortality (7). Anti-AT_1_R and anti-ET_A_R antibodies specifically bound to their respective receptors on endothelial cells, and were shown to induce extracellular signal-regulated kinase phosphorylation, and to increase TGF-β gene expression in endothelial cells, suggesting a possible involvement in fibrosis (7). Interestingly, the same group recently reported activation of human microvascular endothelial cells (HMEC-1) via anti-AT_1_R and anti-ET_A_R antibody-positive IgG from SSc sera (30). The mRNA levels of the pro-inflammatory interleukin-8 (IL-8) and of the vascular cell adhesion molecule-1 (VCAM-1) were increased in HMEC-1 after exposure to anti-AT_1_R and anti-ET_A_R antibody-positive IgG from SSc patients (SSc-IgG). Exposure of HMEC-1 to SSc-IgG increased neutrophil migration through endothelial cells and activation of ROS. Human fibroblasts showed increased expression of type I collagen after treatment with anti-AT_1_R and anti-ET_A_R antibody-positive SSc-IgG. Increased neutrophil counts in bronchoalveolar lavage fluids and structural abnormalities of the lungs of naive mice were also detected after treatment with anti-AT_1_R and anti-ET_A_R antibody-positive SSc-IgG (30).

Finally, a study using cDNA microarrays to evaluate gene expression in SSc dermal fibroblasts demonstrated that several autoantigen genes specifically targeted in SSc (fibrillarin, centromere protein B, centromere autoantigen P27, and RNA polymerase II) were overexpressed. Quantitative RT-PCR confirmed overexpression of these autoantigens and also revealed increased levels of ATA transcripts in SSc fibroblasts (31).

## Concluding Remarks

In summary, serum autoantibodies are considered important biomarkers for the early and accurate diagnosis of SSc, and are associated with distinctive clinical subsets and different prognostic features. Although SSc-related autoantibodies were historically considered to be mutually exclusive and remain stable over the course of the disease, in recent studies using more sensitive immunoassays a more frequent coexistence of autoantibodies in SSc patients than previously appreciated has been reported. Finally, some autoantibodies directed against certain autoantigen targets have been shown to induce inflammation, to activate fibroblasts, to favoring collagen synthesis and deposition, and to activate endothelial cells thereby participating in the pathogenesis of SSc. The elucidation of the pathogenic role of autoantibodies in SSc may identify new therapeutic targets for this challenged disease.

## Conflict of Interest Statement

Cristiane Kayser declares no potential conflict of interest. Marvin J. Fritzler is a consultant to Inova Diagnostics Incorporated (San Diego, CA, USA), he has received gifts in kind from Euroimmun GmbH (Luebeck, Germany), and he is the Director of an autoantibody diagnostic testing laboratory, Mitogen Advanced Diagnostics (Calgary, AB, Canada).
